# A piezo surgery with corticotomies and implant placement as part of a multidisciplinary approach to treat malocclusion disorder in an adult patient: clinical report

**DOI:** 10.1186/s40729-015-0021-3

**Published:** 2015-08-13

**Authors:** Federico Gelpi, Daniele De Santis, Simone Marconcini, Francesco Briguglio, Marco Finotti

**Affiliations:** 1 Via delle Mimose, 8 Colognola ai Colli, 37030 Verona, Italy; 2 Dental and Maxillofacial Department, University of Verona, Verona, Italy; 3 Department of surgery, University of Pisa Ist. Stomatologico, Tirreno, Italy; 4 Messina, Italy; 5 Padova, Italy

**Keywords:** Multidisciplinary approach, Oral implantology, Piezo device, Corticotomies

## Abstract

This clinical report illustrates a multidisciplinary approach for the rehabilitation of a young adult patient affected by a bilateral edentulous space and an anterior deep bite. The patient required orthodontics and surgical corticotomy and implantology (both performed with a piezo device). A multidisciplinary planning approach, including orthodontics, oral and periodontic surgery, and restorative dentistry, has an important role in the final outcome of treatment. In fact a dental class I occlusion has been established only on the right side. The left side could not be restored to an ideal class I relationship due to the pontic prosthesis. The original collapsed right posterior occlusion was corrected. A stable posterior occlusion was established, and the balancing interference was eliminated. Centric relation and centric occlusion were established at the same vertical dimension of occlusion. The cephalometric analysis and clinical aspect at the end of treatment showed that the patient had improvements in overbite and overjet.

Multidisciplinary management, including endodontic and restorative dentistry, periodontics, corticotomy-assisted orthodontics, implants, and prosthetics, was used for a young female patient with multiple missing teeth, anterior deep bite, and a malocclusion with cant of the occlusal plane. The interaction of interdisciplinary specialties and careful treatment planning were required. The patient also benefited esthetically from our effort.

## Background

In the adult patient, the loss of teeth or periodontal support can cause pathologic migration of a single tooth or group of teeth. This can result in the development of median diastema or general spacing of the teeth with or without incisor inclination, rotation, or tipping of the premolars and molars and consequently collapse of the posterior occlusion with decreasing vertical dimension [1].

Regaining the lost interocclusal space is a requirement for successful treatment in these cases. A multidisciplinary approach such as reduction of the overerupted teeth, which may require a combination of endodontic treatment, periodontal surgery, and a fixed prosthesis afterwards; extraction of the overerupted teeth; surgical reconstruction of the edentulous space; and orthodontic intrusion of the extruded teeth has been suggested for regaining the original space [2–5].

Intrusion of the extrusive opposing teeth orthodontically is the most conservative but also the most difficult and long-acting treatment option [5, 6].

The maxillary corticotomy is another available and suggested technique that facilitates orthodontic intrusion.

This clinical report illustrates a multidisciplinary approach for the rehabilitation of a young adult patient affected by a bilateral edentulous space. The patient required orthodontics and surgical corticotomy and implantology (both performed with a piezo device).

## Case presentation

### Diagnosis and etiology

**Fig. 1:**
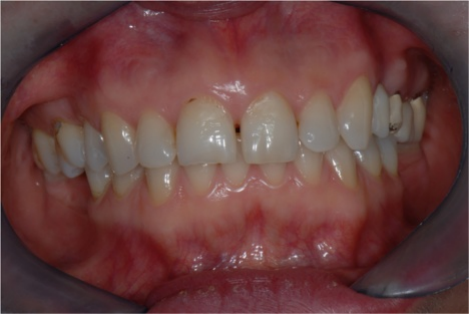
Initial frontal intraoral aspect

**Fig. 2:**
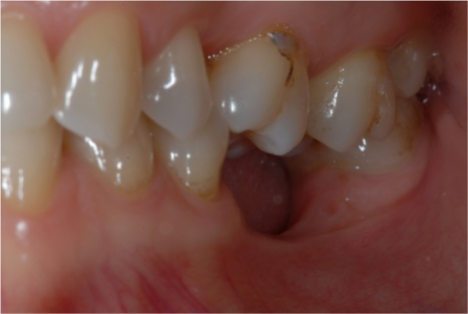
Initial lateral intraoral aspect

**Fig. 3:**
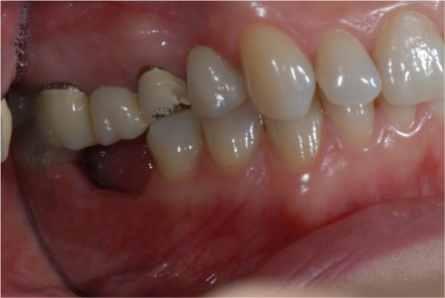
Some metal ceramic crowns in the upper left maxillary arch with a very poor esthetic appearance

A young female patient was referred to our dental clinic to resolve a malocclusion disorder due to missing teeth. She was unsatisfied with the functional aspect of her dentition. She had a second upper right molar very damaged by caries (17) (Figs. 1 and 2); it was also extruded due to missing antagonist teeth (I and II lower right molars 46–47) (Fig. 3). The first upper molar was medially oriented due to a II premolar agenesis. There were some metal ceramic crowns in her upper left maxillary arch with a very poor esthetic appearance. A thorough examination, which included mounted diagnostic casts, was performed.

**Fig. 4:**
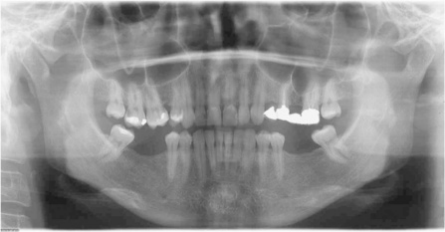
The panoramic radiography and cephalometric analysis revealed a partially edentulous mandible

**Fig. 5:**
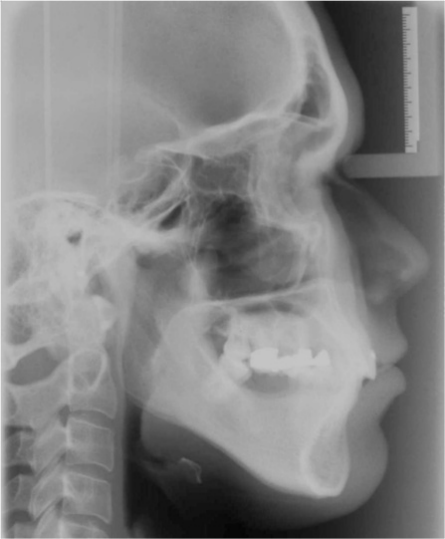
The panoramic radiography and cephalometric analysis revealed a partially edentulous mandible

Clinical examination, panoramic radiography, mounted diagnostic casts, and cephalometric analysis revealed a partially edentulous mandible, and the diagnosis was established: anterior deep bite, a I skeletal class and a III dental class, supraeruption, and drifting and rotation of elements 16 and 17 (Figs. 4 and 5).

Because of her youth, esthetic requirements, and economic opportunities, the patient was advised she could be treated with a multidisciplinary approach to achieve a satisfactory esthetic and functional rehabilitation with restored occlusion.

### Treatment objectives

The following treatment objectives were established for this patient: (1) conservative and endodontic treatment in the teeth damaged by caries; (2) reestablishment of the correct occlusal plane; (3) corticotomy surgery (performed with a piezo device) to accelerate orthodontic-assisted tooth intrusion, alignment, and tipping; and (4) improvement of mouth posterior function through implant surgery to replace missing teeth (performed with a piezo device).

### Treatment progress

After the diagnostic workup was completed, a treatment plan was developed using a specialist team approach involving endodontic, orthodontic, oral surgery, and prosthodontic specialists. The proposed treatments included orthodontic intrusion and tipping of the I and II upper right molars (16 and 17) and uprighting of the lower wisdom teeth and dental alignment.

The first step consisted of endodontic treatment of element 17 and subsequent fiber post buildup and esthetic restoration on elements 11 and 21.

**Fig. 6:**
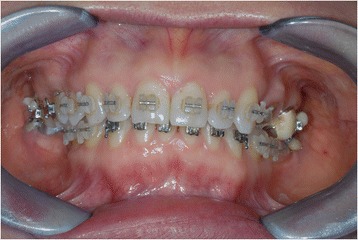
Orthodontic bracket placement: frontal view

**Fig. 7:**
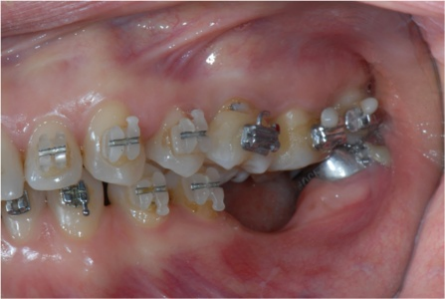
Ortodontic bracket placement: right side view

**Fig. 8:**
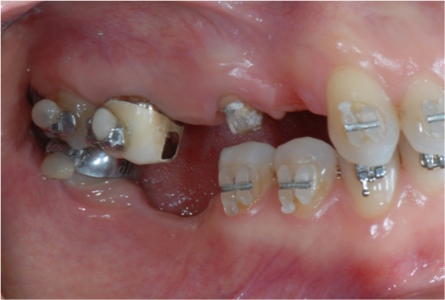
Orthodontic bracket placement: left side view

After that, the 26 pontic removal was planned, and subsequently, orthodontic brackets were placed (Figs. 6, 7, and 8).

This was a preliminary stage before the first surgical corticotomy (performed with a piezo device).

**Fig. 9:**
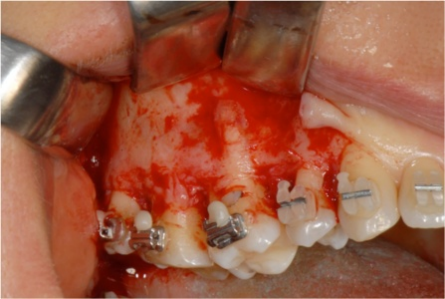
A microsurgical corticotomy was mandatory to assist orthodontic tipping and intrusion of elements 16 and 17

A microsurgical corticotomy was mandatory to assist orthodontic tipping and intrusion of elements 16 and 17. This surgical procedure was performed by a piezo approach (Fig. 9).

**Fig. 10:**
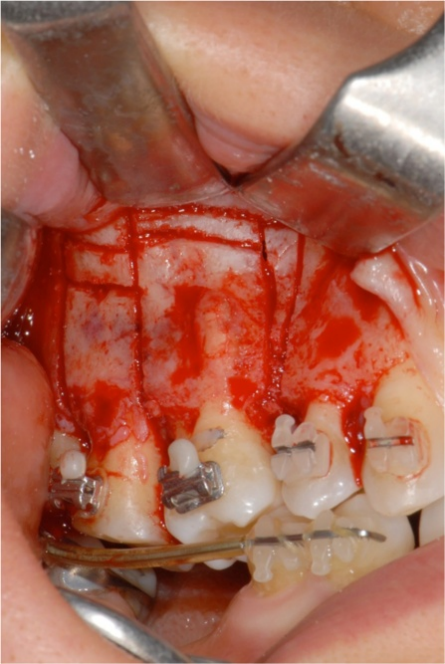
A triangular-shaped corticotomy was performed with inserts OT7 0.55 mm and OT7 special 0.35 mm to accelerate orthodontic tooth movements

A total width flap was elevated to make the cortical subapical and longitudinal bone cut possible. The surgeon had to respect a minimum of 3-mm distance from the apex and 1 mm from the periodontal ligament. A triangular-shaped corticotomy was performed with inserts OT7 0.55 mm and OT7 special 0.35 mm to accelerate orthodontic tooth movements (Fig. 10). The bone cut design was conceived to surgically reduce the amount of bone among root surfaces and assist orthodontic tooth movement.

**Fig. 11:**
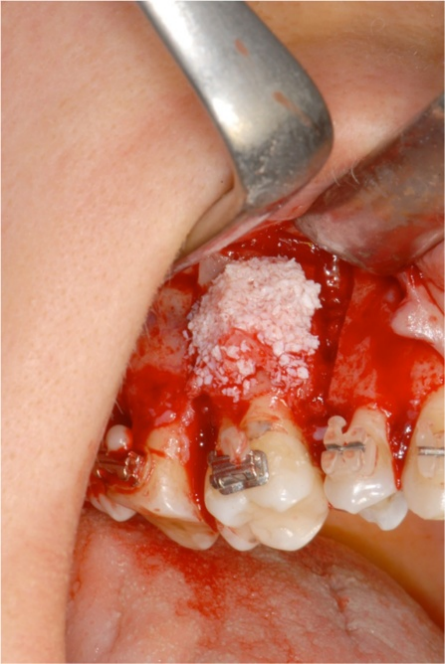
A mesiobuccal root surface exposure of element 16 required bone regeneration through Bio Oss and bone chip application

**Fig. 12:**
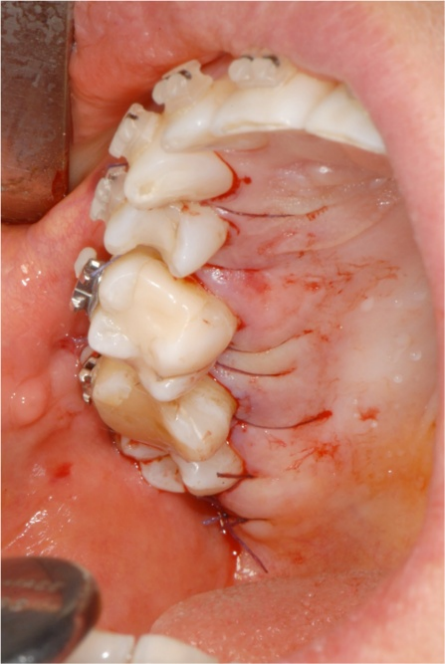
The total width flap was sutured

Moreover, a mesiobuccal root surface exposure of element 16 due to a bone defect was evident. It required bone regeneration through Bio Oss and bone chip application (Figs. 11 and 12).

Orthodontic therapy involved immediate application of strong intrusive forces (>250 g) after corticotomy surgery. A NiTi 18 × 22 diameter archwire was applied to the brackets. It ensured mobilization of the bone maxillary block. The tipping movement of elements 16 and 17 took approximately 12 weeks. The patient was controlled weekly for the first 2 months and twice in the third month. No complications involving the periodontal ligaments or endodontic vessels were observed in the weekly follow-ups.

**Fig. 13:**
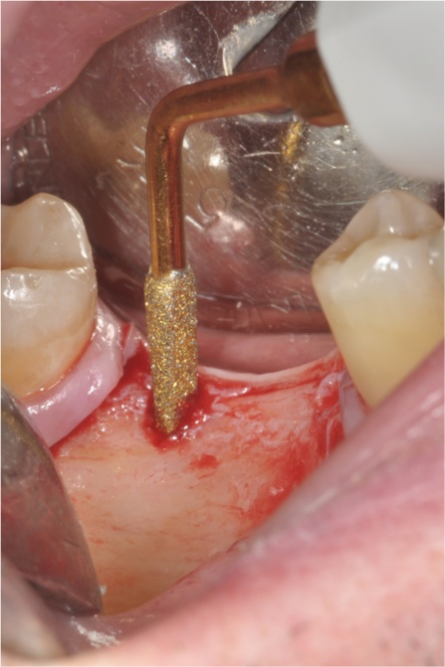
Implant site preparation: OP5, IM2, OT4, and IM3 (correctly in sequence)

**Fig. 14:**
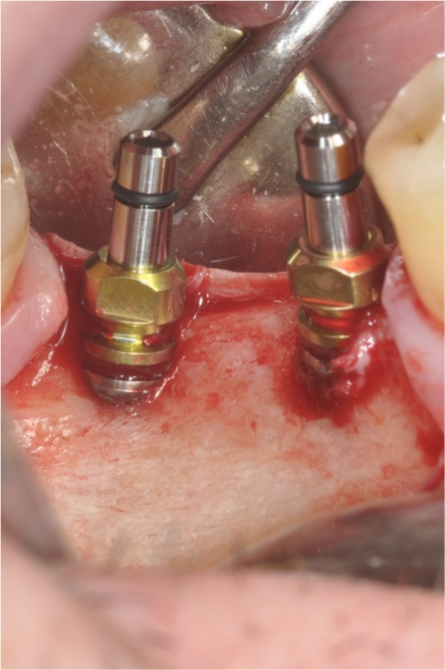
Implants placement after site preparation

**Fig. 15:**
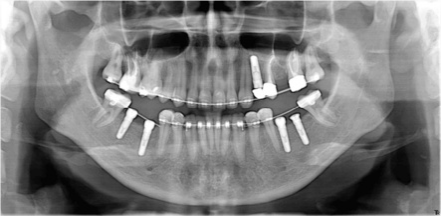
All implants received immediate healing screws

After 7 months, a secondary surgical phase was planned: five Camlog Screw (Line Promote Plus) implants were placed with a minimum 35 N torque in 24 (4.3 mm × 13 mm), 36 (3.8 mm × 13 mm), 37 (3.8 mm × 13 mm), 46 (3.8 mm × 13 mm), and 47 (3.8 mm × 11 mm) sites. This feasible technique provided dedicated inserts for implant site preparation: OP5, IM2, OT4, and IM3 (correctly in sequence) (Figs. 13 and 14) and ensured minimally invasive and stressed implant site preparation. All implants received immediate healing screws (Fig. 15).

**Fig. 16:**
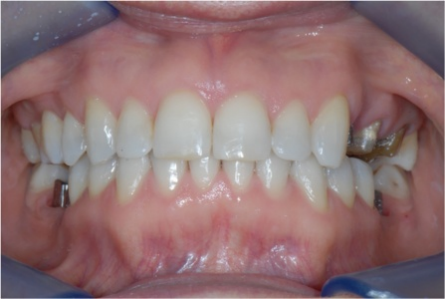
After orthodontic treatment was completed, the prosthodontic phase took place

**Fig. 17:**
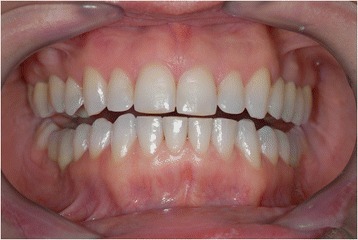
Implants were used for implant-retained prostheses (abutment-cemented crowns), and a three-unit fixed partial denture pontic (crowns 25–27) was placed

**Fig. 18:**
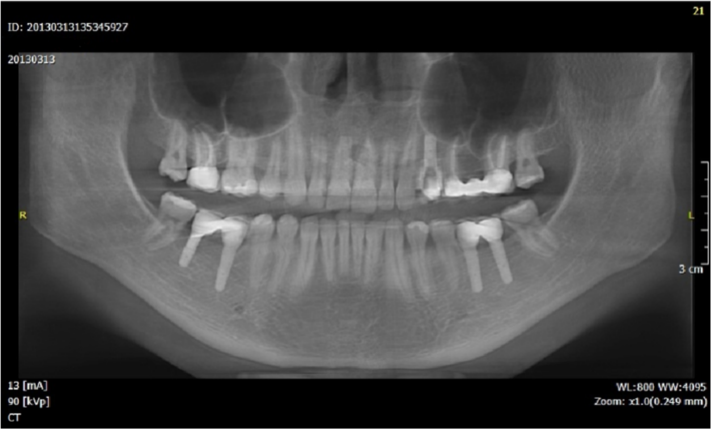
OPT after prosthodontic finalization

The orthodontic treatment took approximately 16 months. After that, the prosthodontic phase took place (Fig. 16). When tooth alignment was completed, all brackets were removed and the definitive restorations were placed. Implants were used for implant-retained prostheses (abutment-cemented crowns), and a three-unit fixed partial denture pontic (crowns 25–27) was placed (Figs. 17 and 18).

### Treatment results

**Fig. 19:**
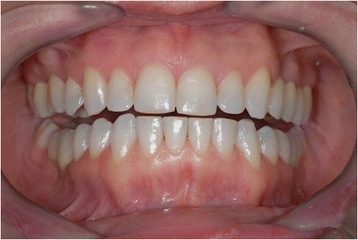
A full-mouth frontal aspect

**Fig. 20:**
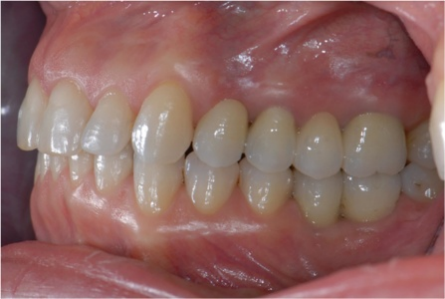
The left side could not be restored to an ideal class I relationship from the original class II due to the pontic prosthesis

**Fig. 21:**
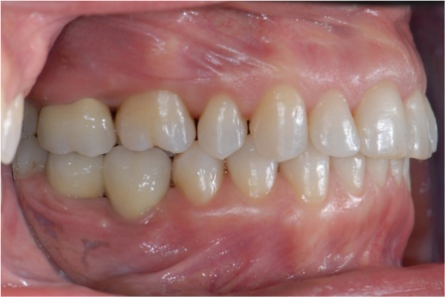
A dental class I occlusion was established only on the right side (lateral aspect)

The left side could not be restored to an ideal class I relationship due to the pontic prosthesis (Figs. 19 and 20). A dental class I occlusion was established only on the right side (Fig. 21). The original collapsed right posterior occlusion was corrected. A stable posterior occlusion was established, and the balancing interference was eliminated. Centric relation and centric occlusion were established at the same vertical dimension of occlusion. The cephalometric analysis and clinical aspect at the end of treatment showed that the patient had improvements in overbite and overjet.

### Discussion

The management of an anterior deep bite requires adequate treatment planning, especially if the clinical condition is associated with posterior DVO (vertical occlusion dimension) reduction due to multiple missing teeth. A multidisciplinary planning approach, including orthodontics, oral and periodontic surgery, and restorative dentistry, has an important role in the final outcome of treatment [7].

In this case, before orthodontic alignment, we decided to intrude elements 16 and 17 (supraeruption and rotation) with a local corticotomy associated with intrusive orthodontic movement. The feasibility of this minimally invasive surgery is strictly linked to the use of a piezo surgery device to perform latero-posterior segmental maxillary osteotomy. Ultrasonic bone-cutting surgery has recently been introduced as a feasible alternative to the conventional tools of cranio-maxillo-facial surgery, due to its technical characteristics of precision and safety.

In fact, it is possible to perform a linear, clean, and thin osteotomic bone cut with maintenance of the integrity of the vascular network. This particular aspect avoids damage of the palatal mucosa and spares use of the chisel to complete the corticotomy. Many studies indicate that conventional cutting tools can produce impairment of pulp blood flow and loss of tooth vitality [8]. Furthermore, vascular compromise can occur due to direct or heat-induced injury to the soft tissue pedicles. To prevent such complications, we support the use of piezoelectric surgery in this critical multipiece surgery, as reported in the literature [9].

In these anatomically difficult conditions, a piezo surgery device provides good intraoperative visibility and a safe and precise osteotomy due to its micrometric characteristics and selective cut [9].

The piezo device offers many versatile inserts; inserts for implant site preparation appear to be particularly useful and versatile. We believe that the piezo surgery device offers many intra- and postoperative advantages and provides desirable clinical outcomes such as a favorable implant success rate, as reported in the literature [10].

In a multidisciplinary treatment approach, a multi-use specific tool such as a piezo device allows simplification of each surgical step within very difficult and complex management planning. In fact, it is possible to reduce intra- and postoperative complications (damage to soft tissues such as nerves, the blood vessel network, and dental pulp), to assist and accelerate intrusive and tipping orthodontic movement of migrated teeth, and to perform safe oral surgery [9].

Moreover, intrusion can be a reliable therapeutic treatment in patients with a healthy periodontal status because it does not result in a decrease of marginal bone level [11].

The best results are obtained when tooth intrusion is performed with light forces (5–15 g) and the line of action of the force passes close to the center of resistance. However, in our clinical report, after corticotomy surgery, tooth intrusion was performed with very high forces (>250 g) to mobilize the bone block, but the final clinical outcomes and periodontal status were satisfactory anyway. In this case, forces did not act on the tooth ligament but on the corticotomized bone: if forces were long-term and intensive on the ligament, hypoxia, root resorption, and vascular damage might occur.

A number of reports have indicated that orthodontic treatment can improve the periodontal situation in patients with pathologic migration by providing good function and improved esthetics after realignment.

It is generally recommended that orthodontic treatment should be preceded by periodontal therapy. In fact, orthodontic treatment when there is an inflammation/periodontal defect can lead to irreversible breakdown of the periodontal system [1].

According to this principle, we decided to cover the root exposure on element 16. Obviously, the corticotomized area was also covered by Bio Oss and bone chips.

This corrective phase was completed before the orthodontic treatment.

Finally, the implant surgery using the piezo device took place. Dental implants have become predictable and reliable adjuncts for oral rehabilitation [12–14].

In this case, no GBR or other sensitive surgical techniques were necessary before or during implant placement.

In our opinion, the piezo device’s versatility offers advantages in implant surgery [9] and improves implant prognosis [10].

A multidisciplinary therapy is usually an expensive and long-term treatment. In this case, the corticotomy performed by the piezo device, as well as precise and flowable planning without any clinical complications, allowed treatment acceleration so it could be tolerated more easily by the patient.

## Conclusions

Multidisciplinary management, including endodontic and restorative dentistry, periodontics, corticotomy-assisted orthodontics, implants, and prosthetics, was used for a young female patient with multiple missing teeth, anterior deep bite, and a malocclusion with cant of the occlusal plane. The interaction of interdisciplinary specialties and careful treatment planning were required. The patient also benefited esthetically from our effort.

The English in this document has been checked by at least two professional editors, both native speakers of English. For a certificate, please see http://www.textcheck.com/certificate/Ml6kvQ.

## Consent

Written informed consent was obtained from the patient for publication of this case report and any accompanying images. A copy of the written consent is available for review by the Editor-in-Chief of this journal.
